# Creating a kidney organoid-vasculature interaction model using a novel organ-on-chip system

**DOI:** 10.1038/s41598-022-24945-5

**Published:** 2022-11-30

**Authors:** Amanda Bas-Cristóbal Menéndez, Z. Du, T. P. P. van den Bosch, A. Othman, N. Gaio, C. Silvestri, W. Quirós, H. Lin, S. Korevaar, A. Merino, J. Mulder, M. J. Hoogduijn

**Affiliations:** 1 Division of Nephrology and Transplantation, Department of Internal Medicine, Erasmus MC Transplant Institute, Erasmus University Medical Center, Molewaterplein 40, 3015 GD Rotterdam, The Netherlands; 2 Department of Pediatrics, Sophia Children’s Hospital, Erasmus University Medical Center, Rotterdam, The Netherlands; 3 Department of Pathology, Erasmus University Medical Center, Rotterdam, The Netherlands; 4 BIOND Solutions B.V., Delft, The Netherlands; 5 Vascular Medicine and Pharmacology, Department of Internal Medicine, University Medical Center, Rotterdam, The Netherlands; 6 Division of Pediatric Nephrology, Department of Pediatrics, Leiden University Medical Center, Leiden, The Netherlands

**Keywords:** Induced pluripotent stem cells, Stem-cell differentiation

## Abstract

Kidney organoids derived from human induced pluripotent stem cells (iPSCs) have proven to be a valuable tool to study kidney development and disease. However, the lack of vascularization of these organoids often leads to insufficient oxygen and nutrient supply. Vascularization has previously been achieved by implantation into animal models, however, the vasculature arises largely from animal host tissue. Our aim is to transition from an in vivo implantation model towards an in vitro model that fulfils the advantages of vascularization whilst being fully human-cell derived. Our chip system supported culturing of kidney organoids, which presented nephron structures. We also showed that organoids cultured on chip showed increased maturation of endothelial populations based on a colocalization analysis of endothelial markers. Moreover, we observed migration and proliferation of human umbilical vein endothelial cells (HUVECs) cultured in the channels of the chip inside the organoid tissue, where these HUVECs interconnected with endogenous endothelial cells and formed structures presenting an open lumen resembling vessels. Our results establish for the first-time vascularization of kidney organoids in HUVEC co-culture conditions using a microfluidic organ-on-chip. Our model therefore provides a useful insight into kidney organoid vascularization in vitro and presents a tool for further studies of kidney development and drug testing, both for research purposes and pre-clinical applications.

## Introduction

Kidney organoids are three-dimensional (3D) structures that, to an extent, resemble the complexity of the human fetal kidney^1^. Kidney organoids contain tissue derived from two embryological niches that give rise to the kidney: the ureteric bud and the metanephric mesenchyme, both derived from the intermediate mesoderm^1^. These embryonic-stage tissues give rise to nephron structures in kidney organoids displaying glomeruli with podocytes (Wilm’s Tumor-1^+^ (WT1^+^)), proximal tubuli (Villin^+^) and distal tubuli (E-cadherin^+^)^2^. Due to their resemblance to native tissue, kidney organoids have proven valuable to better understand human kidney development and study human kidney disease^3^. However, there are several challenges to overcome in the field, including the constrained culturing times and absence of vascularization^3–5^. Kidney organoids lack vasculature, and therefore their culture cannot be sustained over time due to necrotic core formation as a result from insufficient nutrient and oxygen supply in the inner layers of the organoid tissue^6^. This implies that organoids are limited in size and maturation status, and correlate only up to early second trimester embryonic kidney according to transcriptomic analysis^1,7^, which hampers the use of kidney organoids for disease modelling and drug testing^1^. Despite the lack of vasculature, kidney organoids present some CD31^+^ endothelial cells (ECs)^8^. Moreover, high expression of vascular endothelial growth factor (VEGF), a known regulator of vasculogenesis and angiogenesis, has been detected in kidney organoids^9^. Therefore, key components of vascularization are already present in the organoids, but traditional transwell-based protocols fail to achieve perfusable endothelial tissue expansion and maturation. Kidney organoids present EC populations that express specific endothelial maturation markers, particularly melanoma cell adhesion molecule (MCAM, also known as CD146) and platelet endothelial cell adhesion molecule (PECAM, also known as CD31)^10,11^. During development, ECs express MCAM early on, and gain PECAM expression in more mature stages. Eventually, when the endothelium is fully mature, MCAM expression is lost and ECs express only PECAM^10^. Previous research by Halt et al. confirmed that MCAM expression is vital for kidney vasculature development, showing MCAM^+^ cells depletion haltered vascular development whilst PECAM^+^ cells depletion did not, demonstrating that MCAM^+^ cell populations alone are able to rescue endothelial growth^10^. This evidence points towards a crucial role of MCAM^+^ cells in kidney organoid vascularization^11,12^. Despite evidence of the presence of these markers in endothelial populations in kidney organoids^9^, expression of these markers over time are unclear and need to be further investigated to interpret the developmental stage of vascular tissue in kidney organoids.

Regarding vascularization, different approaches have been undertaken to initiate vascularisation of kidney organoids, in particular through implantation in animal models, namely chicken embryos (choriallantoic membrane)^7^ and mice (under kidney capsule)^13,14^. Nevertheless, this vasculature is largely derived from the host tissue, which implies different physiological and immunological properties. In vitro vascularization of organoids is a novel approach that would overcome the problems of xenovascularization. Pseudovascularization of kidney organoids has been described by subjecting them to fluidic shear stress (FSS) in a miniature millifluidic bioreactor^9^. This work shows that fluidic flow acts as a key cue in vascular structure development in kidney organoids, and hypothesizes that the unidirectional flow establishes a VEGF gradient throughout the tissue that gives directionality and polarization to support EC growth^9^. However, further research must be carried out to provide a perfusable vasculature that allows tissue growth and further maturation whilst avoiding necrosis in the core of the organoids. Here we present a novel approach towards vascularization of kidney organoids using an organ-on-chip system: the BIOND microfluidic organ-on-chip with perfusable channels. Organ-on-chip systems constitute a valuable tool for developing vasculature and vascularization models, as their compartmentalization enables differential cell seeding and co-culturing with other tissues. Perfusable endothelial-coated channels have been achieved in different chip models under different protocols using HUVECs^15–18^. HUVECs proliferate and the microvascular structures anastomize with each other and form perfusable lumens^15,16^. Nevertheless, despite the efficiency to generate vascular networks in vitro, when co-cultured with organoids, these models have failed to induce vascular proliferation inside the organoid tissue^18^. Thereby, in this study we aimed to achieve endothelial ingrowth inside kidney organoids via co-culture with synthetic vessels in an organ-on-chip, and thus create an efficient model for organoid vascularization.

## Results

### Microfluidic organ-on-chip system supports kidney organoid development

**Figure 1:**
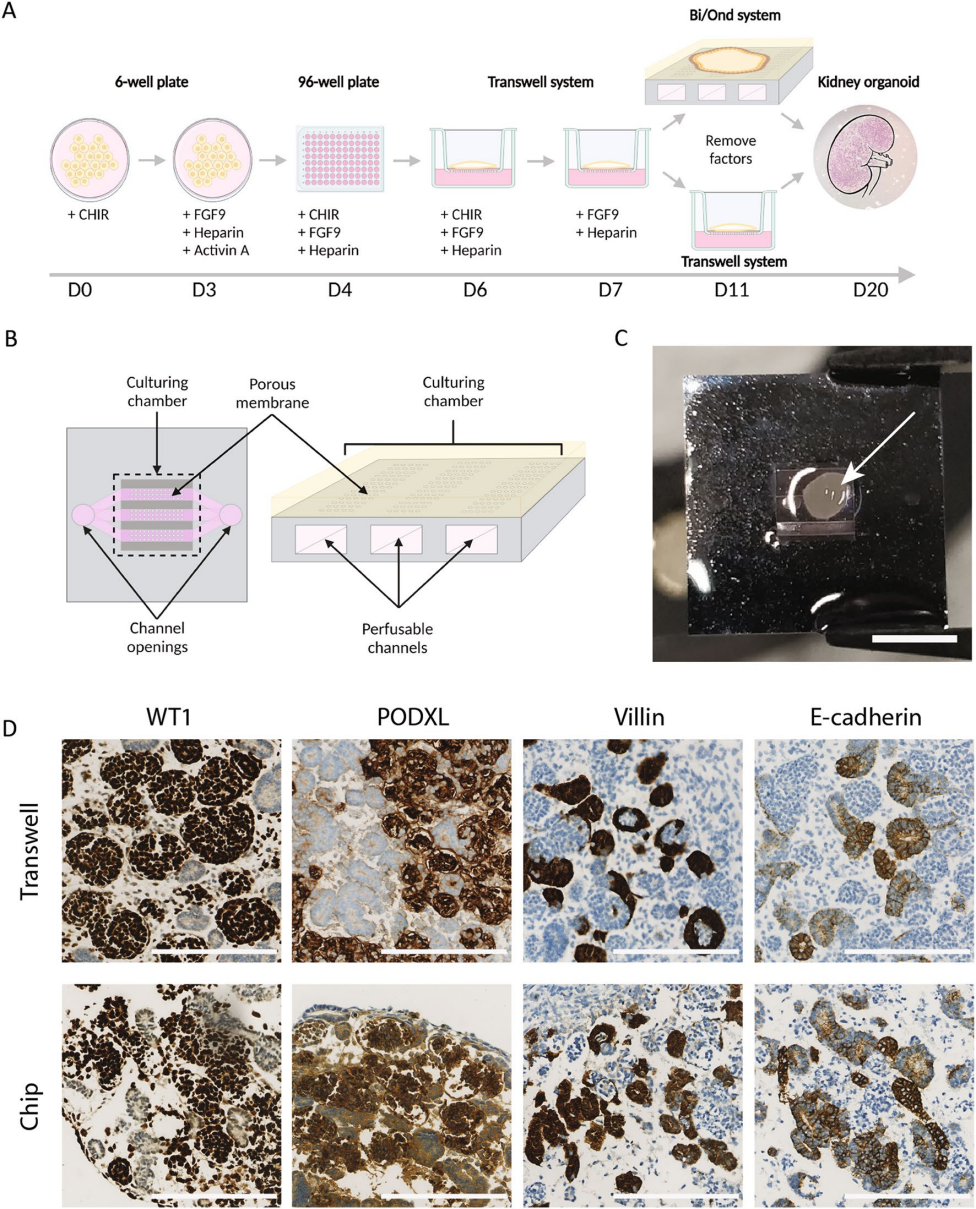
Organoids cultured on chip present all major nephron structures. (**A**) Schematic representation of the organoid generation protocol timeline, indicating days, addition of factors and the different phases of culture. (**B**) Schematic representation of the BIOND chip indicating main parts of the device. (**C**) Kidney organoid on chip (indicated with arrow) at day 20 of the protocol before collection, scale bar = 3 mm. (**D**) Immunohistochemical staining of sections of kidney organoids cultured both on transwell and on chip. Stainings show both organoids present glomeruli (WT1^+^), podocytes (PODXL^+^), proximal tubuli (Villin^+^) and distal tubuli (E-cadherin^+^), scale bar = 200 µm.

Kidney organoids were generated from iPSC of healthy donors over a 20 day differentiation period (Fig. 1A). On day 11 organoids were placed onto the culturing chamber of the organ-on-chip system and subjected to microfluidic flow by pumping of medium through the 3 microfluidic channels beneath. Organoids were harvested at day 20 (Fig. 1A). Organoids cultured on chip are subjected to submerged culturing in the chip system, and medium pumped through the three microfluidic channels reaches the culturing chamber through the porous membrane (Fig. 1B), contrary to transwell systems where the organoid is subjected to an air–liquid interface. Kidney organoids cultured on the chip system (Fig. 1B, C) showed expression of the glomerular marker WT1, podocyte marker PODXL, proximal tubuli marker Villin, and distal tubuli maker E-cadherin similar to organoids cultured on transwell membranes (Fig. 1D). These results confirm culturing of kidney organoids is supported by the microfluidic organ-on-chip system.

### Endothelial tissue in kidney organoids cultured on chip shows directed maturation patterns

**Figure 2:**
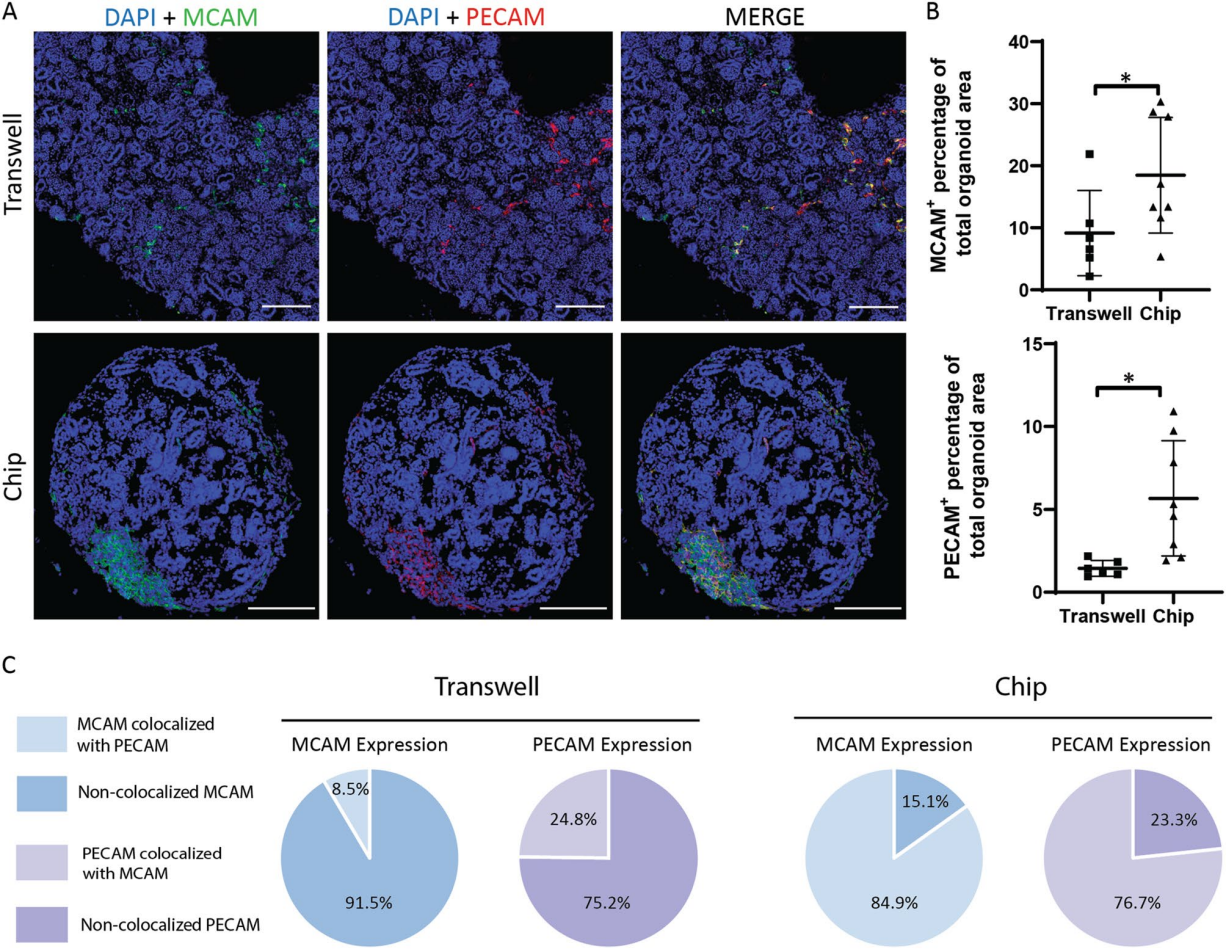
Kidney organoids cultured on chip show expanded EC populations and directed endothelial maturation patterns. (**A**) Immunofluorescent images of DAPI-MCAM-PECAM co-staining showing kidney organoids cultured on transwell and on chip, scale bars = 200 µm. (**B**) Statistical analysis of MCAM and PECAM expression in kidney organoids based on percentage of total area. Organoids cultured on chip *(n* = *8)* showed higher areas positive for both MCAM and PECAM *in comparison to organoids cultured on transwell (n* = *6)*. Each data point = one organoid. * = P < 0.05. Error bar = SD. (**C**) Colocalization analysis showing percentages of colocalized and non-colocalized markers in kidney organoids cultured on transwell and on chip.

To better assess the effects of the chip system on the vasculature, we performed a series of analyses focused on organoid vascular tissue maturation. We performed the analysis based on the area of MCAM^+^ and PECAM^+^ EC populations using immunofluorescent images of MCAM-PECAM co-staining (Fig. 2A). Area analysis showed a two-fold increase of MCAM^+^ and a four-fold increase of PECAM^+^ areas in comparison to organoids cultured on transwell (Fig. 2B). Moreover, colocalization analysis of MCAM and PECAM showed organoids cultured on transwell had a higher percentage of ECs expressing non-colocalized MCAM and PECAM whilst the percentage of cells showing colocalization of both markers was low (Fig. 2C), (Supplementary Fig. 1). Organoids cultured on chip, showed a high percentage of cells co-expressing MCAM and PECAM, whilst the percentage of ECs expressing non-colocalized markers was low (Fig. 2C). This suggests organoids cultured on the chip system present directed maturation of endothelial populations, resembling intermediate maturation stages in which ECs present both MCAM and PECAM markers^10^. Meanwhile, ECs of organoids cultured on transwell present higher percentages of immature and fully mature ECs, which is not coherent with tissue maturation patterns.

### HUVECs create 3D synthetic vessels on chip channels

**Figure 3:**
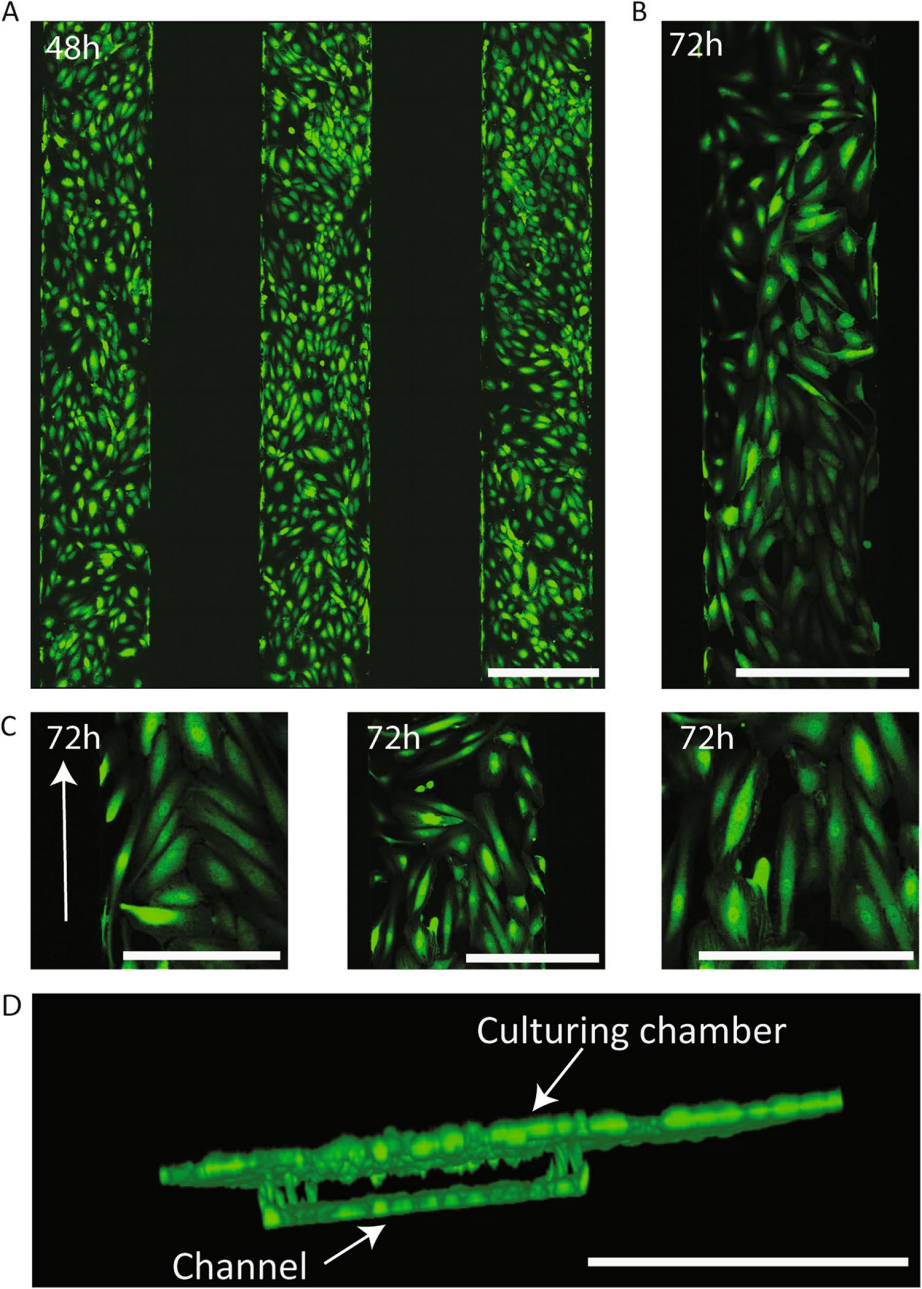
GFP^+^ HUVECs seeded on microfluidic chip channels form synthetic 3D vessels. (**A**) fluorescence image of the chip’s three microfluidic channels after 48 h of static culture, showing GFP^+^ HUVECs forming a monolayer inside these channels. (**B**) Fluorescence image of a chip channel after 48 h of static culture + 24 h of flow, showing HUVECs remained in the channels after exercising fluidic stress and adopted a directionality concurrent with the flow direction. (**C**) Detail images of the chip channels after 48 h of static culture + 24 h of flow, in which the directionality acquired by the HUVECs can be observed (indicated with arrow). (**D**) 3D render of confocal stack of a chip channel lined with GFP^+^ HUVECs showing establishment of a 3D synthetic vessel presenting cells in all planes. Scale bars = 500 µm (**A**, **B**, **D**) and 250 µm (**C**).

We next aimed to induce migration of endothelial cells from the chip channels into the organoids. To do this, HUVECs were cultured in the microfluidic channels. HUVECs formed a confluent monolayer on all surfaces of the chip’s channels after 48 h of static culture (Fig. 3A) (Supplementary video 1, Supplementary video 2). HUVECs were still present in the form of a confluent monolayer 24 h after initiating flow (Fig. 3B, C). This confirmed the capacity of these cells to resist flow sheer stress. Flow was sustained at 3µL/min throughout the experiment, controlled by the flow sensor placed before the chips in the circuit. Upon withstanding flow for 24 h, HUVEC acquired directionality on the channels concurrent with flow direction as it is seen in vascular tissue architecture, suggesting the flow exercised over these microfluidic channels mimics native vascular conditions^19^ (Fig. 3B, C, Supplementary Fig. 2). Moreover, 3D-rendering of the confocal stacks showed that HUVECs not only covered all planes of the channels creating a synthetic vessel, but cells also migrated through the porous membrane and formed a monolayer in the bottom plane of the culturing chamber (Fig. 3D) (Supplementary video 1, Supplementary video 2). This effectively proved the capacity of HUVECs to migrate from the channels towards the culture chamber through the pores present in the membrane (Fig. 3D).

### Co-culture of kidney organoids with endothelialized channels promotes HUVEC migration into the organoid tissue and formation of open lumen structures

**Figure 4:**
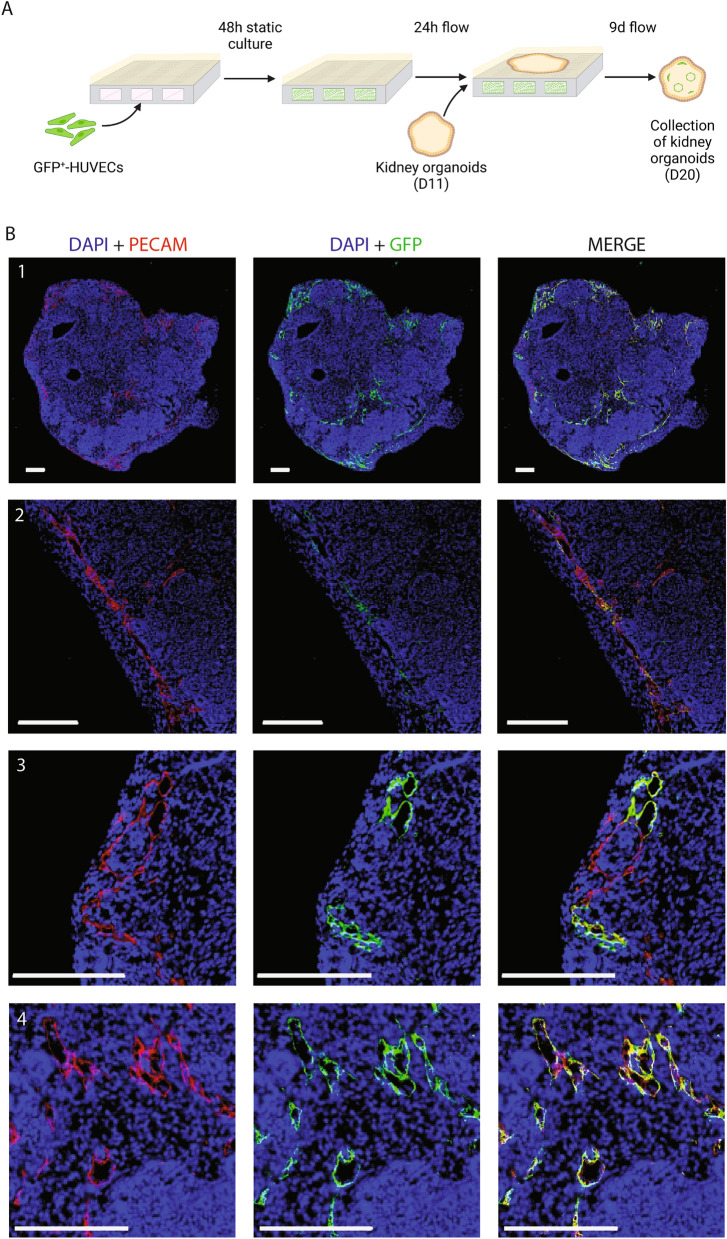
GFP^+^ HUVECs migrate from the chip channels into the organoids and establish integrated vascular structures presenting an open lumen. (**A**) Schematic representation of the co-culture timeline of HUVECs and kidney organoids, indicating static culture, exercise of flow and addition and collection points of kidney organoids. (**B**) Immunofluorescent images of DAPI-PECAM-GFP of organoids co-cultured with GFP^+^ HUVECs on the microfluidic chip system for 9 days. Shown in the images are vascular structures derived from the GFP^+^ HUVECs integrated in the organoid tissue. (**B1**) Whole organoid presenting vascular structures derived from GFP^+^ HUVECs. (**B2**) Detail image of large vascular structure on longitudinal section. Open lumen can be appreciated throughout the structure. (**B3**) Detail image showing continuity of vascular structures formed by HUVECs (PECAM^+^, GFP^+^) with native ECs (PECAM^+^, GFP^-^). (**B4**) Detail image of a transversal cut of several vascular structures with clearly visible open lumens. Scale bars = 200 µm.

To investigate HUVEC migration through the porous membrane we lined the chip channels with GFP^+^ HUVECs and cultured kidney organoids above for 9 days (Fig. 4A). Upon collection of the organoids, organoid tissue contained GFP^+^ HUVECs derived from the channels, forming vascular structures presenting an open lumen (Fig. 4B). Moreover, these GFP^+^ structures connected with endogenous ECs (GFP^-^) from the organoid tissue as shown by PECAM immunostaining (Fig. 4B). We therefore demonstrated the formation of endothelial structures derived from a bio-artificial vessel in kidney organoids in a microfluidic organ-on-chip.

## Discussion

The present study demonstrates that kidney organoids can be successfully differentiated on an organ-on-chip and present all relevant nephron structures upon culture on chip, demonstrating the feasibility of this system for organoid culture and differentiation. Moreover, the implemented system facilitates organoid culturing protocols due to its automatized pump that enables culture without the need for medium changes every 48 h. Organoids cultured on chip showed higher expression of endothelial markers MCAM and PECAM in comparison with organoids cultured on transwell based on the area analysis. Furthermore, upon area colocalization analysis of these markers, we observed that kidney organoids cultured on chip show directed endothelial maturation. Organoids cultured on transwell showed higher expression of both non-colocalized markers, which would correlate with early and late stages of endothelial maturation, suggesting their endothelial populations do not display cohesive native tissue maturation patterns^10^. These results further confirm the fluidic flow supplied by fluidic devices is an important cue for EC maturation as shown in previous work^9,20^. Moreover, the implemented system presents certain advantages over previous devices shown to support kidney organoid culturing, by enabling perfusability of the channels and migration of HUVECs into the tissue that successively form vascular structures. These channels supported the formation of perfusable synthetic vessels upon HUVEC culturing. This established a proof-of-concept for the use of this organ-on-chip to mimic vasculature in vitro. The HUVECs resisted 9 days of fluidic flow shearing stress and adopt a directionality in concordance with native endothelium when subjected to unidirectional flow^19^, further proving the capacity of this endothelialized microchannels to mimic vasculature in vitro. Upon co-culture with the channels endothelialized with GFP HUVECs, organoid tissue presented GFP^+^ HUVECs forming vascular structures that connected to native organoid ECs. These results demonstrate that co-culture of kidney organoids and HUVECs on the chip system leads to endothelial cell infiltration and vascularization under microfluidic flow. To our knowledge, we present the first successful infiltration of HUVECs inside kidney organoids where they contribute to vascular development. Our research constitutes a first step towards functional in vitro vascularization of kidney organoids. We hypothesize the high production of VEGF by the kidney organoids (Supplementary Fig. 3) acts as a powerful cue for HUVECs’ migration towards the tissue. This claim is supported by various publications reporting the role of VEGF to recruit ECs^8,21–23^, although additional factors may play an important role in HUVEC migration. These factors need to be further researched, as well as the mechanism that leads HUVECs to form vascular structures that are integrated with native organoid EC populations. Functional vascularization of organoids has previously been achieved in vivo by implantation in animal models, and has proved to aid in the maturation processed of kidney organoids^8,13,14^. These results confirm vascularization can improve kidney organoid maturation. However, reliance on animal models hinders the use of these models, as the vasculature derives largely from host tissue^14^, which hampers translatability of results in regards to human tissue. Ultimately, our goal is to transition from in vivo models of vascularisation to an in vitro model using an organ-on-chip based system, which allows us to generate a fully human-derived research model that can be perfused. In the future, we expect to further improve this model by optimizing the use of iPSCs-derived ECs to obtain a model for kidney organoid-vasculature interaction that presents the same genetic background and that could pose as a powerful tool for personalized medicine with applications such as patient-specific drug testing. The implemented system presents a series of limitations regarding organoid culture that need to be overcome through future optimization processes. Although we were able to obtain successful kidney organoids upon culture on chip, our protocol still relies on generating the organoids following traditional transwell protocols^7^. Therefore, the organoids are only subjected to flow for part of the differentiation period. Moreover, the system largely constrains the number of organoids that can be generated during one differentiation protocol, which slows down the data obtention process. Regarding the volume of medium necessary to culture each organoid, the use of a non-circulating medium set-up significantly increases the volume used, and therefore the cost/organoid. Finally, we must take into account a clear limitation of the kidney organoid field being reproducibility^4^. Although improved protocols to generate kidney organoids have emerged, reproducibility still poses as a challenging issue in the field^2,24^, meaning large variability of the results. A collaborative effort must be done in this regard amongst researchers in the field to ultimately achieve consistently reproducible kidney organoids.

## Summary

Kidney organoids derived from human iPSCs are a powerful model to study kidney development and disease, however their lack of vascularization hampers their maturation and longevity. In this work we employed a novel organ-on-chip system to initiate vascularisation in kidney organoids. We proved that this organ-on-chip system supports kidney organoid differentiation and improves the number of endogenous endothelial cells as well as their maturation patterns. Moreover, we created synthetic vessels by seeding HUVECs in the microfluidic channels of the chip. When co-cultured with kidney organoids in the chip system, HUVECs from the channels were able to migrate through the porous membrane and establish vascular-like structures in the organoid tissue, which presented open lumens and connected to endogenous endothelial cells. This research demonstrates the first steps of in vitro vascularization of kidney organoids, which is of great value for a diverse range of applications such as developmental studies and drug testing.

## Materials and methods

All methods were carried out in accordance with relevant guidelines and regulations. A list of reagents used in this research can be found in Supplementary table 1.

### iPSC culturing

Human primary skin fibroblasts were obtained from two healthy donors after written informed consent, and reprogrammed to iPSC using a single, multicistronic lentiviral vector encoding OCT4, SOX2, KLF4, and C-MYC 19 at the Erasmus MC iPSC core facility. Cell collection and experimental procedures were reviewed and approved by the medical ethics committee of the Erasmus University Medical Centre under project number MEC-2017-248. Frozen human iPSCs were thawed and cultured in Essential 8 medium (E8) (Thermo Fisher Scientific, Massachusetts, USA) on Geltrex (Gibco, NL). The medium was refreshed daily and cells passaged in clumps upon reaching 70–80% confluency, using 0.5 mM ethylenediamine tetraacetic acid (EDTA) (Invitrogen, Netherlands) at split ratios of 1:4 to 1:12 depending on confluency and growth rates. Cells were washed twice with Dulbecco’s phosphate buffered saline (DPBS) previous to EDTA addition. Morphology, growth rates and differentiation were closely monitored during the culturing process to ensure pluripotency of iPSCs.

### Organoid culturing

iPSC-derived kidney organoids were generated based on the protocol designed by Garreta et al.^7^ (Fig. 1C). iPSCs were dissociated into single-cells via incubation for 5 min using TrypLE™ Select (Invitrogen, Netherlands) at 37 °C. Cells were plated as a monolayer on a 6 well-plate and E8 medium was supplemented with Rock inhibitor RevitaCell (Invitrogen). After 24 h medium was replaced with advanced RPMI 1640 basal media (Invitrogen) supplemented with 8 µM CHIR99021 (Sigma-Aldrich, Missouri, USA). After 3 days of 8 µM CHIR99021 treatment, medium was replaced with advanced RPMI 1640 basal media supplemented with 200 ng/mL FGF9 (Prepotech, United Kingdom), 1 µg/mL heparin (Sigma-Aldrich) and 10 ng/mL activin A (R&D systems, Minnesota, USA). On day 4 of the differentiation protocol cells were detached from the plate surface using TrypLE™. Cell suspensions were diluted to a final concentration of 5 × 10^6^ cells/mL in advanced RPMI 1640 basal media supplemented with 3 µM CHIR99021, 200 ng/mL FGF9 and 1 µg/mL heparin and 0.5 × 10^6^ cells were seeded in each well of a V-bottom 96 well plate (non-extra low attachment) (Thermo Fisher Scientific). The 96 well plate was centrifuged at 300 g for 3 min to form cell pellets. After 48 h the cell pellets were carefully placed on 6-well plate transwell membranes and 1.2 mL of advanced RMPI 1640 basal media with 3 µM CHIR, 200 ng/mL FGF9 and 1 µg/mL heparin were added to the base of each transwell to generate an air–liquid interphase culture condition. After 24 h, medium was replaced with advanced RPMI 1640 basal media containing 200 ng/mL FGF9 and 1 µg/mL heparin, and organoids were cultured for 48 h without media changes. Medium was then refreshed, and afterwards growth factors were removed by replacing the medium with advanced RPMI 1640 basal media without further additions. Medium was refreshed every other day for the next 9 days. On the last day of the protocol, organoids were collected and fixed in a 4% paraformaldehyde (PFA) solution, stained using a 1:100 tissue marking dye kit (Thermo Fisher Scientific) in DPBS solution and embedded in 1% agarose (Roche, Switzerland) before proceeding with immunohistochemistry and immunofluorescence.

### HUVEC culturing

Human umbilical cord endothelial cells expressing GFP constitutively were commercially obtained (product code ZHC-2402, Cellworks, San Francisco, USA). Cells were grown in EBM-2 endothelial cell growth basal medium (Lonza, Switzerland) containing 5% heat inactivated foetal bovine serum. Cells were passaged upon reaching 80–90% confluence using Trypsin–EDTA solution (Sigma-Aldrich) and incubation at 37 °C for 3 min. Cells were kept under passage 10 to ensure proliferative and angiogenic capabilities. The GFP expression of this commercial line was validated using flow cytometry and WT-HUVEC as negative control. Briefly, cells were detached using Trypsin–EDTA solution, resuspended in the appropriate volume of sheath fluid (Thermo Fisher Scientific) and analysed using the BD FACSCanto™ II Flow Cytometry Systems (BD biosciences, Belgium) (Supplementary Fig. 4).

### Organ-on-chip system

We acquired the BIOND organ-on.chip system (inCHIPit™ and comPLATE™) (BIOND Solutions B.V., Delft, The Netherlands). The inCHIPit™ is produced using polydimethylsiloxane film supported by a silicon frame, and consists of a culture chamber that communicates with the underlying three 400 µM-wide channels through 4 µM-wide pores^25^ To generate flow, chips are placed on the comPLATE™ and this is connected to a pressure pump that generates 800 mbar of pressure, and pumps circulating medium from the reservoir through the tubing and towards the chip channels, producing an estimated flow rate across the porous membrane of 0.2 µL/min. The system allows to maintain a constant flow thanks to the pressure regulator placed in the system, which ensures continuous fresh medium and stable shear stress. These perfusable channels of the chip system allow us to maintain organoid culture subjected to fluidic flow, which is a key factor in itself for vasculature development^9,20^. The chips were subjected to plasma ashing for 2 min using the plasma cleaner PDC-32G-2 (Harrick Plasma, New York, USA) at high radio frequency setting equivalent to 18 W. After treatment, chips were sterilized with 70% EtOH and stored submerged in MilliQ water to avoid contact with air. Previous to cell seeding, chips were cleaned in 70% EtOH for 5 min followed by two cleaning steps of 5 min with DPBS. Thereafter, a 0.01% fibronectin solution was injected on the chip channels and incubated for 1 h at 37 °C. Channels were flushed twice with DPBS before seeding of the HUVEC to remove any excess fibronectin.

### HUVEC seeding on chip channels

HUVECs were removed from their culture flasks by trypsinisation and centrifuged at 800 g for 5 min. The pellet was resuspended in EBM-2 medium at a concentration of 2 × 10^6^ cells/mL. Empty 200 mL filter tips (Greiner Bio-One, NL) were placed at one end of every chip before cell seeding. Using the same tips, 100 µL of cell suspension was flushed through the channels using positive pressure. The upper chamber of the system was filled with 150 µL of EBM-2 medium. The tips were left on the chip system and the plate was incubated at 37 °C with 5% CO2 for 1 h to allow the HUVECs to attach to the channel surface. After one hour, tips were removed and replaced with new tips, one of which containing 200 µL of EBM-2 medium. The flow generated by passive medium flow towards the empty tip at the other end of the channel ensured the removal of unattached cells and cell debris. HUVECs were subjected to static culture for 48 h to ensure proper attachment and proliferation, replenishing the medium in the tips and the upper chamber after 24 h. After 48 h of static culture, the chips were connected to the flow setup. After static culture, the complate was attached to pressure-based flow control, Flow EZ™ (Fluigent, Germany). Microfluidic flow was applied at a constant rate of 3µL/min for 48 h before placement of the kidney organoids in the top chamber. Flow and pressure were monitored by using the AIO software in combination with a flow sensor (Fluigent, Germany) and a bubble trap was added to minimise bubbles. Pressure threshold was set at 200 mbar to avoid leakage. Imaging of the channels was performed using confocal imaging, tiled images and stacks. Stacks were rendered into a 3D figure using the image analysis software Fiji. To confirm directionality of the HUVECs after flow, we manually counted the number of cells in comparable portions of the channels before (48 h) and after flow (24 h flow, 72 h total). To assess directionality cells that showed an elongation and direction concurrent with the flow direction with a deviation not greater than 45° were considered as having acquired directionality.

### On chip culturing of kidney organoids

Organoids were collected after 5 days culture in the transwell system and placed on the top well of the chip system. Two organoids were placed in each chip. Medium of the reservoir was changed to 30% EBM-2 + 70% advanced RPMI 1640 basal media to support co-culture conditions of kidney organoids and HUVECs. Flow rate was kept at 3 µL/min and organoids were collected after 9 days for fixation and processing.

### Quantitative real-time PCR

RNA from day 20 kidney organoids was isolated with Roche High Pure RNA Isolation Kit (Roche). First-strand complementary DNA (cDNA) was synthesized from 400 ng RNA in 80 μL reaction volume using Moloney Murine Leukemia Virus Reverse Transcriptase Kit (M-MLV RT; Invitrogen), random primers (Promega), and RNasin® Ribonuclease Inhibitor (Promega). Real-time PCR quantification of gene expression was measured using TaqMan Gene Expression Master Mix (Applied Biosystems, Lithuania), TaqMan Gene Expression Assays (Life Technologies) and a StepOnePlus Real-Time PCR System instrument (Applied Biosystems, Singapore)**.**

### Immunohistochemical staining

Four-micron sections of formalin-fixed paraffin-embedded organoids were stained with haematoxylin and eosin according to manufactures instructions (Ventana Medical Systems Inc., Arizona, USA). Immunohistochemistry was performed with an automated, validated and accredited staining system (Ventana Benchmark ULTRA, Ventana Medical Systems) using ultraview or optiview universal DAB detection Kit. In brief, following deparaffinization and heat-induced antigen retrieval the tissue samples were incubated according to their optimized time with the antibody of interest (Supplementary table 2). Incubation was followed by haematoxylin II counter stain for 12 min and then a blue colouring reagent for 8 min according to the manufacturer’s instructions (Ventana Medical Systems Inc., Arizona, USA). Healthy kidney was used as positive control tissues (Supplementary Fig. 5).

### Multiplex immunofluorescent staining

Co-staining of CD31 and CD146 with was performed by automated multiplex IF using the Ventana Benchmark Discovery (Ventana Medical Systems Inc., Arizona, USA). In brief, following deparaffinization and heat-induced antigen retrieval with CC1 (#950-500, Ventana) for 32 min the tissue samples were incubated firstly with anti-CD31 for 32 min at 37 °C followed by detection with Red610 (#760-245, Ventana). Antibody denature step was performed using CC2 (#950-123, Ventana) for 8 min at 100 °C. Secondly, anti-CD146 was incubated for 32 min at 37 °C followed by detection with FAM (#760-243, Ventana). Slides were washed in DPBS and covered with DAPI in vectashield. Antibody information and clonality can be found in Supplementary Table 2. All slides used in this project were randomly picked before imaging, and orientation of the organoids was undetermined after processing.

### Computational image analysis

Quantitative Assessment of marker expression in kidney organoid sections was performed using the image processing software Fiji^26^. Expression area was calculated by delimitation of each individual organoid. Expression of each marker was calculated as a relative percentage of the total area. Colocalization analysis was performed with the Fiji plugin JACoP^26,27^ for the markers MCAM and PECAM. Threshold was determined individually using Costes' automatic threshold^27,28^. Statistical processing of the results was performed using two-tailed unpaired t-test with Welch’s correction with a confidence interval of 95% using GraphPad Prism version 8.0.0 for Windows (GraphPad Software, San Diego, California USA, www.graphpad.com).

## Supplementary Information

Supplementary Information 1.

Supplementary Information 2.

Supplementary Information 3.

## Data Availability

The datasets used and/or analysed during the current study are available from the corresponding author on reasonable request.

## Abbreviations

3D: Three-dimensional
DPBS: Dulbecco’s phosphate buffered saline
EBM: Endothelial basal medium
EC: Endothelial cell
EDTA: Ethylenediamine tetraacetic acid
FACS: Fluorescence-activated cell sorting
FSS: Fluidic shear stress
GFP: Green fluorescent protein
HE: Haematoxylin–Eosin
HUVEC: Human umbilical vein endothelial cell
iPSC: Induced pluripotent stem cell
MCAM: Melanoma cell adhesion molecule
PECAM: Platelet endothelial cell adhesion molecule
PFA: Paraformaldehyde
VEGF: Vein endothelial growth factor
WT1: Wilms’ tumor-1
WT: Wild type
