# Supplementary material for: Creating a kidney organoid-vasculature interaction model using a novel organ-on-chip system
Source: Sci Rep. 2022 Nov 30;12:20699. doi: 10.1038/s41598-022-24945-5 (PMC9712653; doi:10.1038/s41598-022-24945-5)
Supplement: Supplementary file 1 — Supplementary Information 1. [file 41598_2022_24945_MOESM1_ESM.docx]

Supplementary material


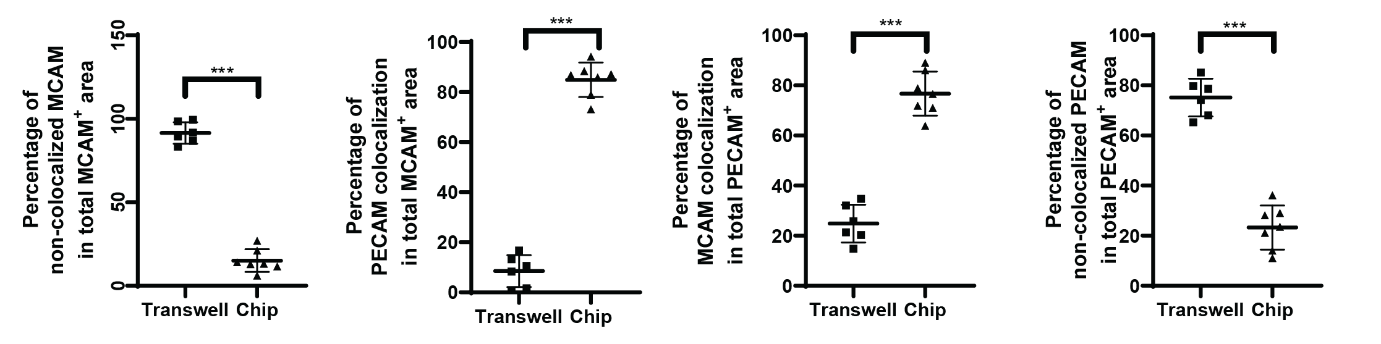


**Supplementary Figure 1: colocalization analysis of MCAM and PECAM expression.** Colocalization analysis of MCAM and PECAM in organoid endothelial cells. Organoids cultured on chip (n = 7) show higher percentages of colocalization for both markers, whilst organoids cultured on transwell (n = 6) show higher percentages of non-colocalization. Each data point = 1 organoid. Error bars = SD. *** = P < 0.001.


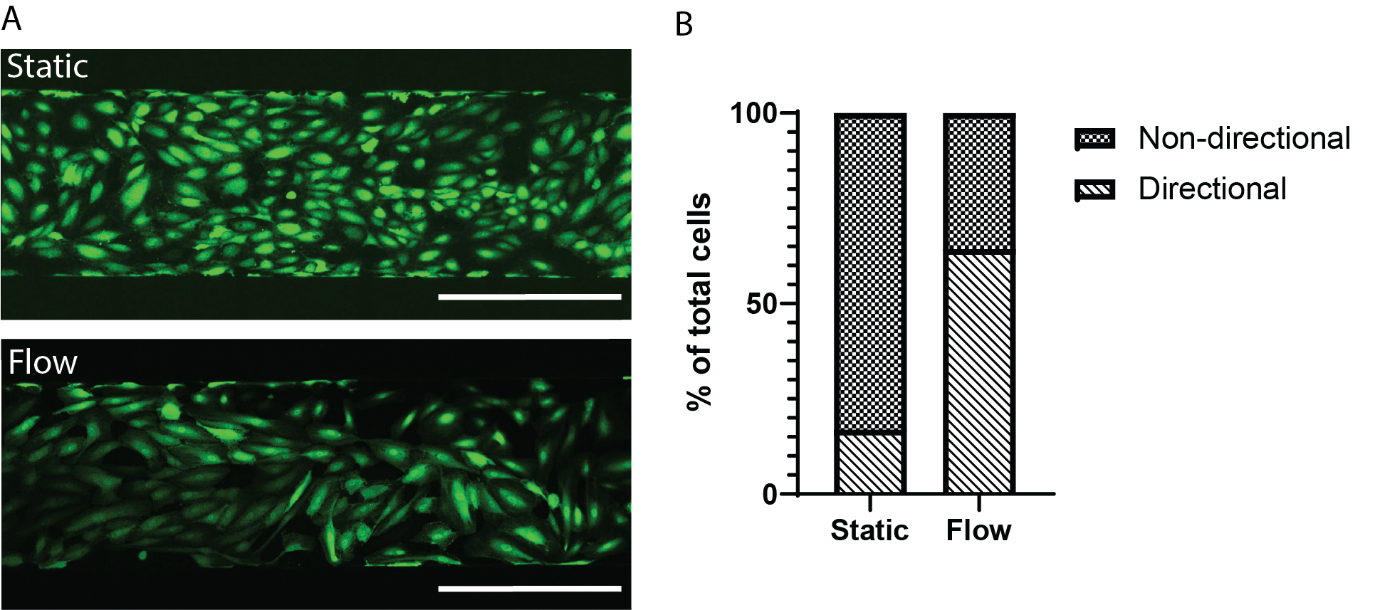


**Supplementary Figure 2: HUVECs acquire directionality upon start of flow conditions.** A) Portions of the chip channels imaged with confocal microscopy after 48h of static culture (top) and after 48h of static culture + 24h of flow (bottom). Scale bar = 400 µm. B) Graphical representation of the percentage of directional vs. non-directional cells in both static and flow conditions.


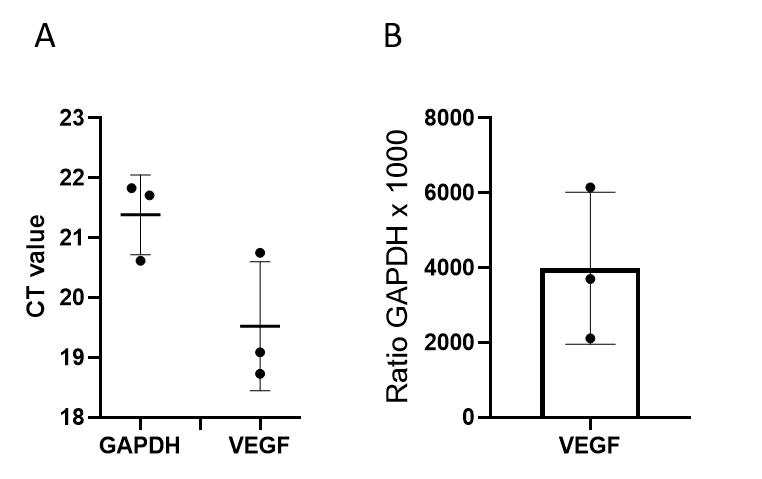


**Supplementary Figure 3: VEGF mRNA expression of kidney organoids.** A) CT (cycler threshold) values of housekeeping gene GAPDH compared to CT values of VEGF. B) VEGF mRNA expression normalized to GAPDH. Each data point = 1 organoid, error bar = SD.


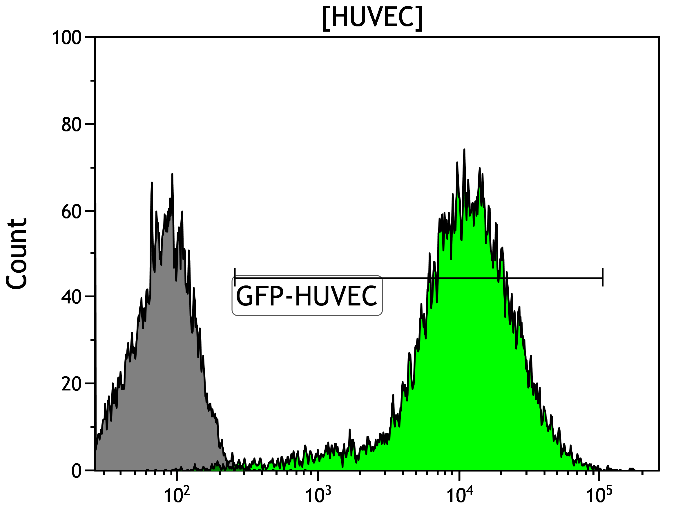


**Supplementary Figure 4: validation of GFP expression in HUVECs:** Flow cytometry results showing >99% of GFP-HUVEC expressed GFP (green peak). Grey peak indicates WT-HUVEC.


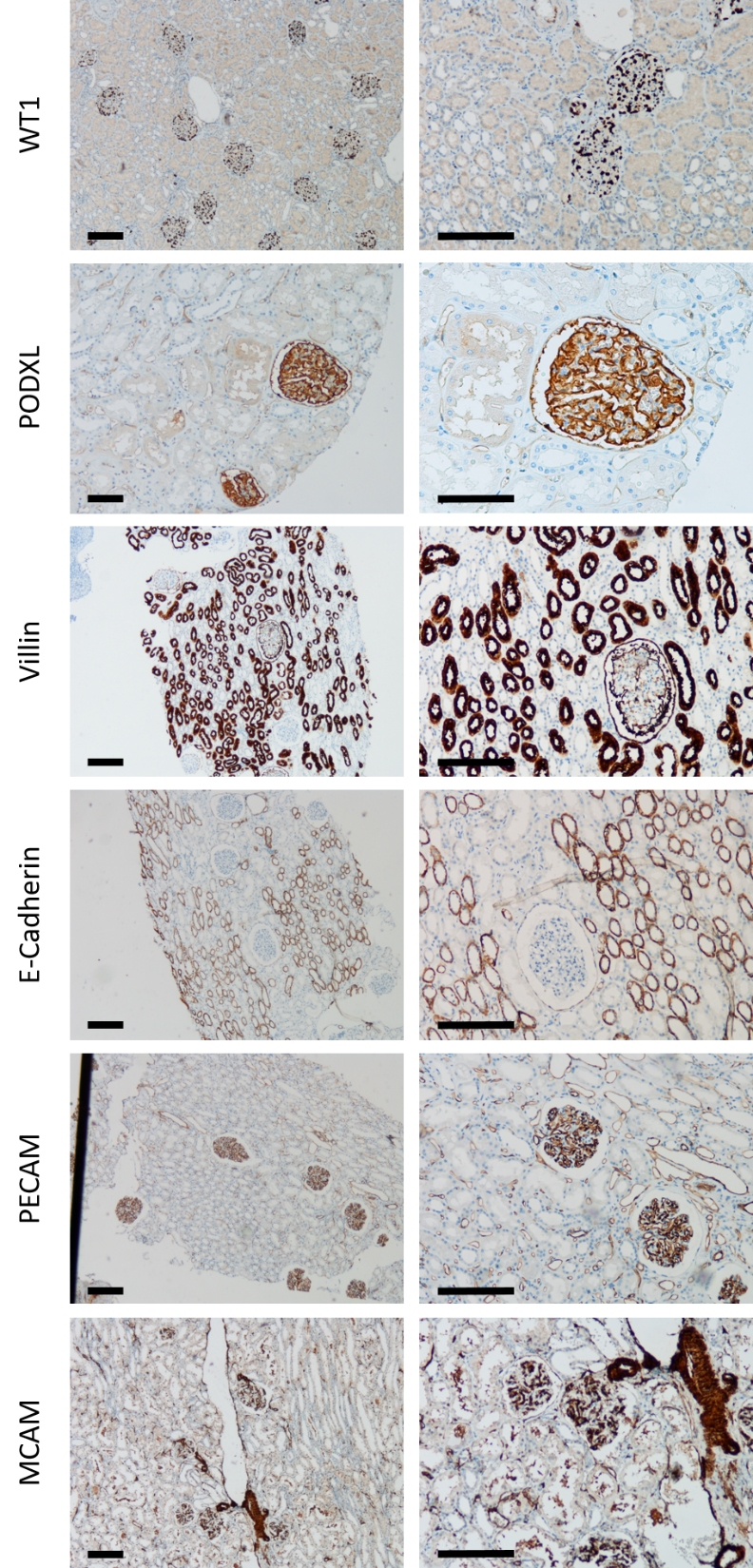


**Supplementary Figure 5: immunohistochemical staining controls in healthy human kidney tissue.** Bright field images of immunohistochemical stainings of the markers WT1, PODXL, Villin, E-Cadherin, PECAM and MCAM on healthy human kidney tissue used to validate organoid stainings. Scale bars = 200 µM.

## **
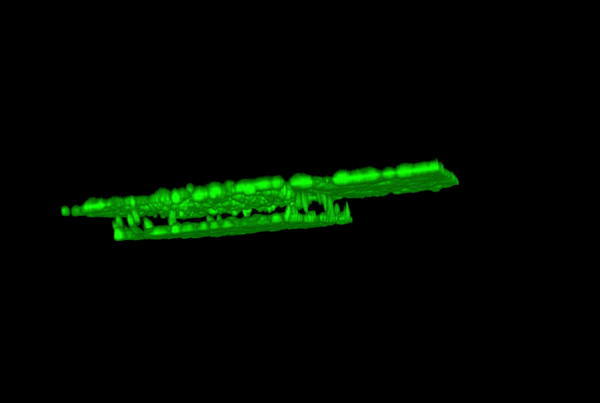
**

## **Supplementary video 1: 3D render of endothelialized chip channel I.** 3D figure rendered from confocal tiled stacks rotating along Y axis. GFP^+^ HUVECs can be seen in all planes of the chip channel 48h after seeding. Channel width = 500 µm.

## **Supplementary video 2: 3D render of endothelialized chip channel II.** 3D figure rendered from confocal tiled stacks rotating along X axis. GFP^+^ HUVECs can be seen in all planes of the chip channel 48h after seeding. Channel width = 500 µm.

## **Supplementary Table 1: Reagents**

| Product | **Company** | **Catalogue number** |
| --- | --- | --- |
| Activin A | R&D Systems | 338-AC-010/CF |
| Advanced RPMI 1640 Medium | Invitrogen | 1263/3020 |
| Agarose | Roche | 11685660001 |
| CHIR99021 | Tocris Bioscience | 4423/10 |
| EBM-2 | Lonza | 190860 |
| EDTA | Invitrogen | 155750020 |
| Tissue marking kit | Thermo Fisher Scientific | [C1311](https://www.thermofisher.com/order/catalog/en/US/adirect/lt?cmd=catProductDetail&entryPoint=adirect&productID=C1311&messageType=catProductDetail) |
| Essential 8 basal medium | Thermo Fisher Scientific | [A1517001](https://www.thermofisher.com/order/catalog/product/A1517001) |
| FGF9 | Prepotech | 100-23 |
| Fibronectin | Sigma-Aldrich | FC010 |
| Geltrex | Invitrogen | [A1413201](https://www.thermofisher.com/order/catalog/product/A1413201) |
| GFP-HUVECs | Cellworks | ZHC-2402 |
| Heparin | Sigma-Aldrich | [H0200000](https://www.sigmaaldrich.com/NL/en/product/sial/h0200000?context=product) |
| Lipofectamine stem | Life Technologies | [STEM00001](https://www.thermofisher.com/order/catalog/product/STEM00001) |
| DPBS | Invitrogen | 10010023 |
| PFA | Klinipath | 4078-9001 |
| Primer GAPDH | Thermo Fisher Scientific | Hs 999999.m1 |
| Primer VEGF | Thermo Fisher Scientific | Hs 00173626.m1 |
| RevitaCell | Invitrogen | [A2644501](https://www.thermofisher.com/order/catalog/product/A2644501) |
| RPMI 1640 | Invitrogen | [61870036](https://www.thermofisher.com/order/catalog/product/61870036) |
| Transwells | Corning | CLS3450-24EA |
| Trypan blue | Invitrogen | [15250061](https://www.thermofisher.com/order/catalog/product/15250061) |
| Tryple E select | Invitrogen | [12605036](https://www.thermofisher.com/order/catalog/product/12605036) |
| Trypsin-EDTA | Sigma-Aldrich | T3924-100ML |
|  |  |  |

**Supplementary Table 2. Immunohistochemistry information**

| **Antibody** | **Type** | **Concentration** | **Company** | **Clone** | **Procedure** | **Ab incubation**  **time** |
| --- | --- | --- | --- | --- | --- | --- |
| Villin | Anti-Rabbit | 1/800 | Abcam | EPR3491 | Optiview CC1 32' | 32 minutes |
| ECAD | Anti-Mouse | 0.314 µg/ml | Ventana | 36 | Optiview CC1 32' | 16 minutes |
| PODXL | Anti-Rabbit | 1/1000 | Abcam | EPR9518 | Ultraview CC1 32' | 32 minutes |
| WT1 | Anti-Mouse | 2.49 µg/ml | Cell Marque | 6F-H2 | Optiview CC1 56' | 32 minutes |
| CD31 | Anti-Mouse | 0.4 µg/ml | Ventana | JC-70 | Ultraview CC1 32' | 28 minutes |
| CD146 | Anti-Mouse | 1/1000 | Origene | UMAB155 | Optiview CC1 32' | 32 minutes |
