# Supplementary figures and images for: Creating a kidney organoid-vasculature interaction model using a novel organ-on-chip system
Source: Sci Rep. 2022 Nov 30;12:20699. doi: 10.1038/s41598-022-24945-5 (PMC9712653; doi:10.1038/s41598-022-24945-5)

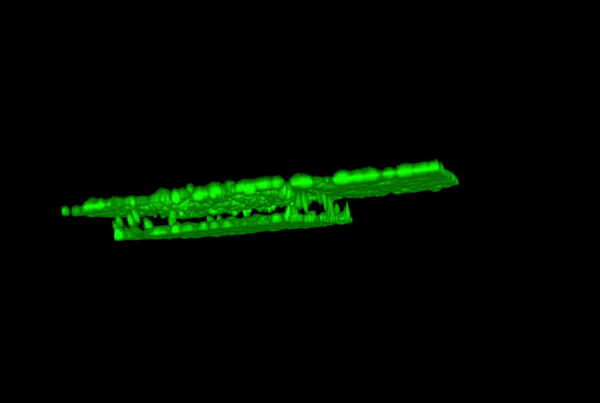

Supplement: Supplementary file 2 — Supplementary Information 2. [file 41598_2022_24945_MOESM2_ESM.gif]

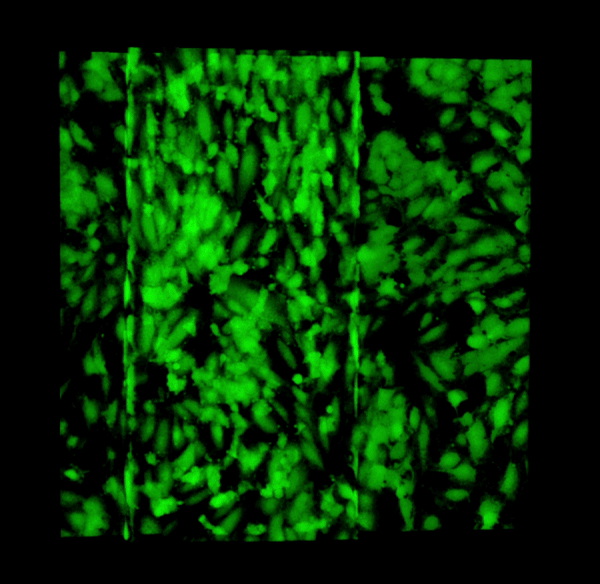

Supplement: Supplementary file 3 — Supplementary Information 3. [file 41598_2022_24945_MOESM3_ESM.gif]
